# Transcriptional analysis of the innate immune response of ducks to different species-of-origin low pathogenic H7 avian influenza viruses

**DOI:** 10.1186/1743-422X-10-94

**Published:** 2013-03-23

**Authors:** Michele N Maughan, Lorna S Dougherty, Lauren A Preskenis, Brian S Ladman, Jack Gelb, Erica V Spackman, Calvin L Keeler

**Affiliations:** 1 Department of Animal and Food Sciences, University of Delaware, Newark, DE 19716, USA; 2 Southeast Poultry Research Laboratory, USDA-ARS, 934 College Station Road, Athens, GA 30605, USA

**Keywords:** Duck, Innate immunity, Pathogenesis, Avian influenza, Low pathogenicity, Transcriptome, Spleen, qRT-PCR, Avian innate immunity microarray (AIIM)

## Abstract

**Background:**

Wild waterfowl, including ducks, represent the classic reservoir for low pathogenicity avian influenza (LPAI) viruses and play a major role in the worldwide dissemination of AIV. AIVs belonging to the hemagglutinin (H) 7 subtype are of epidemiological and economic importance due to their potential to mutate into a highly pathogenic form of the virus. Thus far, however, relatively little work has been conducted on elucidating the host-pathogen interactions of ducks and H7 LPAIVs. In the current study, three H7 LPAIVs isolated from either chicken, duck, or turkey avian species were evaluated for their comparative effect on the transcriptional innate immune response of ducks.

**Results:**

Three H7 LPAIV isolates, chicken-origin (A/chicken/Maryland/MinhMa/2004), duck-origin (A/pintail/Minnesota/423/1999), and turkey-origin (A/turkey/Virginia/SEP-67/2002) were used to infect Pekin ducks. At 3 days post-infection, RNA from spleen tissue was used for transcriptional analysis using the Avian Innate Immune Microarray (AIIM) and quantitative real-time RT-PCR (qRT-PCR). Microarray analysis revealed that a core set of 61 genes was differentially regulated in response to all three LPAIVs. Furthermore, we observed 101, 135, and 628 differentially expressed genes unique to infection with the chicken-, duck-, or turkey-origin LPAIV isolates, respectively. qRT-PCR results revealed significant (p<0.05) induction of IL-1β, IL-2, and IFNγ transcription, with the greatest induction observed upon infection with the chicken-origin isolate. Several key innate immune pathways were activated in response to LPAIV infection including the toll-like receptor and RIG-I-like receptor pathways.

**Conclusions:**

Pekin ducks elicit a unique innate immune response to different species-of-origin H7 LPAIV isolates. However, twelve identifiable genes and their associated cell signaling pathways (RIG-I, NOD, TLR) are differentially expressed regardless of isolate origin. This core set of genes are critical to the duck immune response to AI. These data provide insight into the potential mechanisms employed by ducks to tolerate AI viral infection.

## Background

The study of host pathogen interactions between ducks and avian influenza virus (AIV) is vital to an understanding of the global transmission of avian influenza (AI). The two pathotypes of AI – low pathogenicity (LP) and high pathogenicity (HP) are classified based on their pathogenicity in chickens and the amino acid sequence at the hemagglutinin cleavage site [1]. Of particular interest are the H5 and H7 subtypes of AIV, the two hemagglutinin subtypes that have historically mutated from the LP to HP forms [2].

The experiments described herein were part of a larger study published by Spackman *et al.*[3] in which the pathogenesis of 12 North American H7 LPAIV isolates were evaluated in three avian species: specific pathogen free (SPF) white leghorn chickens (*Gallus gallus domesticus*), broad breasted white turkeys (*Meleagris galopova*) and Pekin ducks (*Anas platyrhynchos domesticus*). The Spackman *et al.*[3] study concluded that the severity of disease and the degree of virus shed relied on specific combinations of species and isolates. Additionally, they concluded that turkeys may be more susceptible to clinical disease from the H7 LPAI than either chickens or ducks.

This report expands the previous study to examine the transcriptional response of ducks to LPAIV. Our 4,959 element avian innate immunity microarray (AIIM) has been successfully used to evaluate the transcriptomic response of several avian species to various microbial challenges, including ducks and avian influenza [4]. In the present study, we utilized the AIIM to characterize the global host immune response of ducks to three H7 low pathogenicity avian influenza (LPAIV) isolates. The aim of this study was to evaluate the consequences of H7 LPAIV infection in ducks with viruses isolated from chickens, ducks, and turkeys.

To elucidate the host mechanisms employed in response to LPAIV infection, we evaluated gene expression changes of the natural host (ducks) to different isolates of LPAIV. We hypothesized that the species-of-origin of an isolate would induce different gene expression patterns related to the innate immune response in Pekin ducks. Gene expression in response to LPAIV infection has been studied in duck: peripheral blood mononuclear cells (PBMC) [5], lung cell cultures [6], intestine [7], and lung, spleen, and lymphatic tissues [8]. In support of the growing research interest in the duck transcriptional immune response, Crowley *et al.*[9] performed a proof-of-concept microarray study of Pekin ducks infected with high pathogenicity avian influenza virus (HPAIV) H5N1 (A/MuscovyDuck/Vietnam/453/2004).

Adams *et al.* studied the effects of an H11N9 LPAIV on duck PBMC [5]. In their studies, they noted consistent up-regulation of interleukin 6 (IL6), interferon-alpha (IFNA), interferon gamma (IFNG), and interleukin 2 (IL2) at 8, 24, and 36 hours post-infection (hpi), minimal gene expression changes in toll-like receptor 7 and MHC I and II gene expression (<3.0 fold), and down-regulation of interleukin 1-beta (IL1B). The authors concluded that the cytokine responses demonstrate a skew towards a weak Th1 response in duck PBMC and the absence of signs of disease in ducks correlated with low pro-inflammatory cytokine levels. Additionally, Adams *et al.* concluded that, in comparison to the chicken response to LPAIV, the lower overall expression of IFNs by duck PBMC in response to AIV infection results in a longer viral shedding duration (persistence) and weaker viral clearance.

Fleming-Capua *et al.* 2011 [8] studied the duck splenic immune response to LPAIV (A/mallard/BC/500/05 (H5N2)) and observed no gene expression changes in cytokines important in the signaling and extravasation of dendritic cells and naïve lymphocytes to secondary lymphoid tissues (CCL19 and CCL21). This finding led the authors to conclude that ducks experience a weakened adaptive immune response to LPAIV versus HPAIV.

Our study compares immune related gene expression of ducks infected with different species-of-origin LPAIV isolates.

## Results

### Pathogenesis of LPAIV in Pekin ducks

Clinical disease signs, depression, anorexia, neurological signs, and death, were not observed in Pekin ducks infected with any of the three LPAIV isolates from days 2 through 14 days post-infection (d.p.i.). Three days after infection with LPAIV, three birds from each treatment group were sampled for detection of gross and microscopic lesions. Microscopic lesions were observed in ducks infected with the chicken-origin virus (CK/MD/MinhMa), specifically in the respiratory tract with one bird having rare heterophils in the nasal cavity and rare mucoheterophilic infiltrate in the lumen of a secondary bronchus. Another bird had luminal detritis and multifocal mucosa-associated lymphoid tissue (MALT) hyperplasia in the nasal cavity and patchy cilial loss while the third bird had focal and minimal seroheterophilichistiocytic serositis of the kidney [10]. Microscopic lesions were also noted in ducks infected with the duck-origin (PT/MN/423/99) LPAIV. Specifically, Pekin ducks displayed heterophils in the sloughing or desquamating surface epithelium of the nasal cavity in two of three birds with one of these birds having a focal peracute hemorrhage in the endocardium of the heart while the third bird had no significant lesions. Finally, microscopic lesions were also noted in ducks infected with turkey-origin (TK/VA/67) virus. One duck exhibited pulmonary lesions of bacteria containing heterophilic granulomatous exudate, another bird showed surface bacterial growth on edematous eroding mucosal epithelium in the nasal cavity, and the third bird showed no significant lesions [10]. While lesions were noted in most of the H7 LPAIV-infected ducks, there were no statistically significant differences in gross lesions among the LPAIV isolates.

### Viral shed

**Figure 1:**
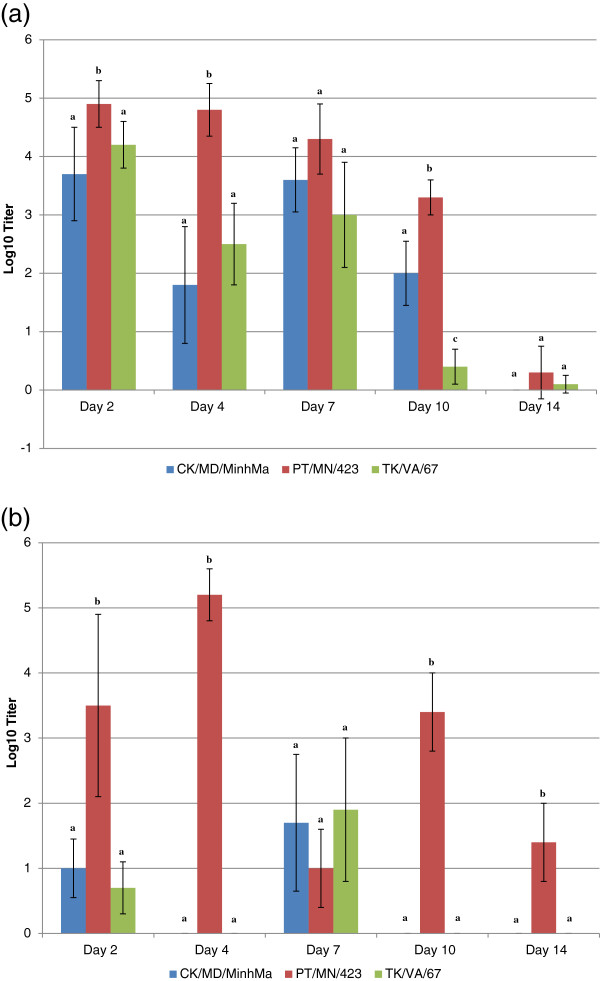
**a.) Mean oral-pharyngeal (OP) virus titers from Pekin ducks.** OP virus titers by day post-infection as determined by quantitative real-time RT-PCR for the influenza M gene [3]. Average qRT-PCR titers expressed as exponents (e.g. a titer value of 4.2 is 10^4.2^). Error bars indicate standard error of titers. Different superscript letters represent statistically significant differences among titers within a given day as determined by a one-way ANOVA and Tukey-Kramer MSD (p<0.05). **b**.) Mean cloacal (CL) virus titers from Pekin ducks. CL virus titers by day post-infection as determined by quantitative real-time RT-PCR for the influenza M gene. Average qRT-PCR titers expressed as exponents (e.g. a titer value of 4.2 is 10^4.2^). Error bars indicate standard error of titers. Different superscript letters represent statistically significant differences among titers within a given day as determined by a one-way ANOVA and Tukey-Kramer MSD (p<0.05).

Absolute quantification qRT-PCR was performed by Spackman *et al.*[3] in order to quantify the amount of virus genomic material (AIV matrix gene) present in the OP and CL swabs and determine viral shed and relative viral titers. The duration of viral shedding was used to determine viral persistence, that is, how long each virus isolate was maintained within the sampled areas (oral-pharyngeal or cloacal). The three LPAIV isolates in this experiment demonstrated different virus recovery and persistence characteristics. As shown in Figure 1, the duck-origin LPAIV (PT/MN/423/99) virus had the highest recovery in the OP swabs throughout the experiment, while the chicken-origin (CK/MD/MinhMa) and turkey-origin (TK/VA/67) viruses did not display significantly different virus shedding (except on day 10). Significant differences (p<0.05) among the virus isolates were observed in persistence and recovery when examining CL swabs, as shown in Figure 1. There was both greater recovery and longer persistence of the duck-origin LPAIV virus (PT/MN/423/99) with virus being recovered throughout the 14 day time course, when compared to the chicken- and turkey- origin viruses, in which virus recovery was only demonstrated on days 2 and 7 post-infection.

### Modulation of gene expression in ducks by LPAIVs

**Figure 2:**
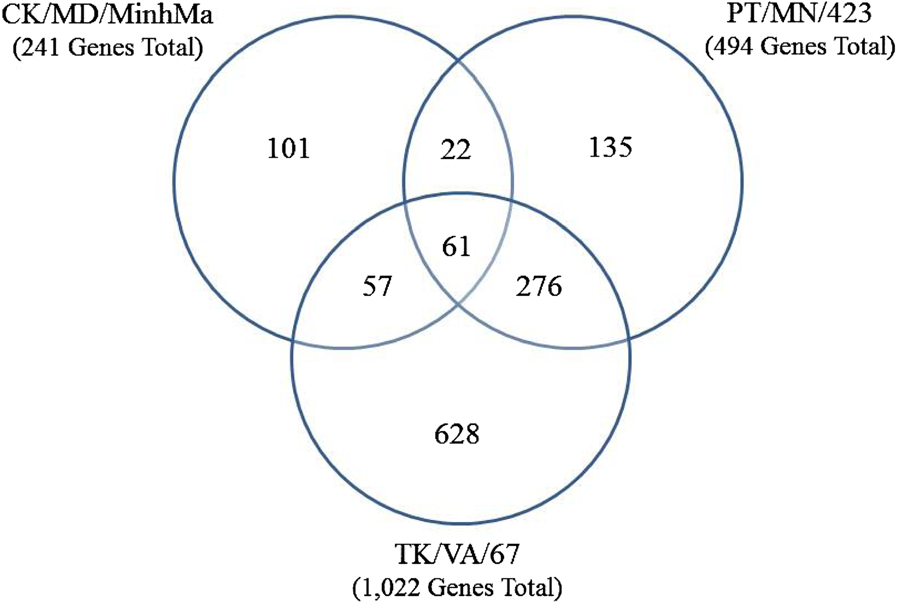
**Genes displaying a two-fold change in expression in response to infection with LPAIV isolates derived from chickens, ducks, and turkeys.** The universe is all genes that were detected in two of three replicates on each AIIM slide in each of the experimental condition slides (3,697 genes total). 1,757 genes are differentially regulated (up- or down-regulated) at least 2 fold over the pooled control samples.

At 3 d.p.i. spleens were harvested and RNA was extracted for use in a microarray experiment utilizing the AIIM. To characterize the transcriptional immune response to LPAIV, we analyzed all of the two-fold differentially regulated genes in each of the three LPAIV infections to find genes unique to a specific species-of-origin isolate or common to all isolates. Combining all three LPAIV-infected treatment groups, there was more down-regulation (1198) than up-regulation (559) of duck splenic genes. There were 101, 135 and 628 2-fold differentially expressed genes unique to infection with the chicken-, duck-, and turkey-origin LPAIV isolates respectively (Figure 2). The number of elements that were up- or down-regulated in response to infection with the chicken-origin virus (CK/MD/MinhMa) was approximately evenly distributed between up- and down-regulated genes (108 and 133, respectively). Additionally, infection with CK/MD/MinhMa yielded the smallest number of differentially expressed genes (241/1757, or 14% of the differentially expressed genes). The number of elements that were down-regulated (352) in response to infection with the duck-origin virus (PT/MN/423) was greater than the number of up-regulated elements (142). The proportion of differentially expressed genes responding to the duck-origin virus (PT/MN/423) was 28% (494/1,757). Finally, the greatest number of differentially expressed genes (1,022) were observed in response to infection with the turkey-origin virus (TK/VA/67), comprising 58% of all differentially expressed genes. Furthermore, 70% (712/1,022) of these differentially expressed genes were down-regulated, and only 30% were up-regulated (310).

### Gene list and gene ontology analysis

Gene Ontology (GO) analysis was conducted in order to examine overall trends in the microarray data, and the subset of differentially expressed genes common to all three LPAIV infections. To identify the biological pathways activated in response to LPAIV infection, we submitted a total of 1,757 Entrez Gene IDs (1,198 2-fold down- and 559 2-fold up-regulated) to GORetriever to obtain GO IDs. GORetriever output was then analyzed in DAVID’s functional annotation tools. Out of the 1,757 genes, 621 genes had DAVID IDs and 10 statistically significant (p<0.05) canonical signaling pathways found in the Kyoto Encyclopedia of Genes and Genomes (KEGG). Functional analyses of the GO terms associated with these gene lists revealed differences in KEGG pathways that were either stimulated or repressed in response to LPAIV infection (Table 1).

**Table 1:** Functional gene ontology annotation using DAVID

**Up-regulated**			**Down-regulated**		
**Pathway**	**%**	**P value**	**Pathway**	**%**	**P value**
gga04142:Lysosome	3.32	0.0155	gga04520:Adherens junction	2.97	0.0000
gga00190:Oxidative phosphorylation	3.32	0.0420	gga04060:Cytokine-cytokine receptor interaction	2.97	0.0456
gga04620:Toll-like receptor signaling pathway	2.90	0.0213	gga03010:Ribosome	2.75	0.0004
gga04621:NOD-like receptor signaling pathway	2.07	0.0259	gga04514:Cell adhesion molecules (CAMs)	2.54	0.0153
			gga04142:Lysosome	2.33	0.0300
			gga04350:TGF-beta signaling pathway	1.91	0.0483

The gene list containing the 559 up-regulated and 1198 down-regulated differentially expressed genes in duck spleen common to all three LPAIV infections at 3 dpi was entered into the DAVID functional annotation software. The following KEGG pathways are enriched in the dataset. The percentage column indicates percentage of differentially expressed genes that mapped to the DAVID database with a corresponding significance value (p<0.05).

To analyze the commonality of the innate immune response amongst all three LPAIV infections, we compared differentially expressed (2 fold up- or down-regulated) genes from each infection and identified the union of these gene lists. Sixty-one genes were differentially expressed in response to all three LPAIV infections (Figure 2), indicating that ducks differentially regulated the same 61 genes regardless of the H7 LPAIV avian-origin isolate. Due to the current completeness of annotation of the chicken genome and the mammalian-bias in functional annotation software, of the 61 differentially expressed genes, our bioinformatics analysis identified 13 genes for functional annotation.

Functional analyses of the GO terms analyzed in DAVID are summarized in Table 2. AIIM data confirms a consistent, amongst all three species-of-origin LPAIV isolate infections, down-regulation of JUN (jun oncogene) and PMM2 (phosphomannomutase 2). JUN is a key regulator of several innate immune pathways and PMM2 functions in several metabolic pathways. The 13 genes were categorized according to their representation in one or more canonical KEGG pathways. Of the 13 genes, 69% (9/13) belong to innate immune pathways illustrating an unsurprising enrichment of genes involved in the immune response to avian influenza. Evidence exists for an association between influenza infection and the subsequent differential regulation of several genes in our list, such as cadherin 1 [11], ATPase [12], mago-nashi homolog [13], proteasome 26S subunit [14], and ribosomal protein L35a [15].

**Table 2:** Functional gene ontology annotation using DAVID

**Gene name**	**KEGG pathway**
ATPase, H+ transporting, lysosomal 16kDa, V0 subunit c	gga00190:Oxidative phosphorylation, gga04142:Lysosome
F-box protein 4	gga04120:Ubiquitin mediated proteolysis
cadherin 1, type 1, E-cadherin (epithelial)	gga04514:Cell adhesion molecules (CAMs), gga04520:Adherens junction
erbb2 interacting protein	gga04621:NOD-like receptor signaling pathway
etoposide induced 2.4 mRNA	gga04115:p53 signaling pathway
interleukin 12B (natural killer cell stimulatory factor 2, cytotoxic lymphocyte maturation factor 2, p40)	gga04060:Cytokine-cytokine receptor interaction, gga04620:Toll-like receptor signaling pathway, gga04622:RIG-I-like receptor signaling pathway, gga04630:Jak-STAT signaling pathway
jun oncogene	gga04010:MAPK signaling pathway, gga04012:ErbB signaling pathway, gga04310:Wnt signaling pathway, gga04510:Focal adhesion, gga04620:Toll-like receptor signaling pathway, gga04912:GnRH signaling pathway
mago-nashi homolog, proliferation-associated (Drosophila)	gga03040:Spliceosome
phosphomannomutase 2	gga00051:Fructose and mannose metabolism, gga00520:Amino sugar and nucleotide sugar metabolism
proteasome (prosome, macropain) 26S subunit, non-ATPase, 7 (Mov34 homolog)	gga03050:Proteasome
ribosomal protein L35a	gga03010:Ribosome
secreted phosphoprotein 1 (osteopontin, bone sialoprotein I, early T-lymphocyte activation 1)	gga04510:Focal adhesion, gga04512:ECM-receptor interaction, gga04620:Toll-like receptor signaling pathway
thioredoxin reductase 1	gga00240:Pyrimidine metabolism

The gene list containing the 61 differentially expressed genes in duck spleen common to all three LPAIV infections at 3 d.p.i. was entered into the DAVID functional annotation software. The following genes and their cognate KEGG pathways are enriched (up- and/or down-regulated) in our dataset.

### Gene expression modulation by LPAIVs (qRT-PCR)

Since the AIIM is a chicken-transcriptome-based microarray used in a cross-species hybridization experiment and a more qualitative than quantitative tool, qRT-PCR using primers derived from duck-specific gene sequences was performed. Select publicly available duck immune gene sequences were analyzed using qRT-PCR to obtain quantitative levels of gene expression of interferon-α (IFNA), interferon-β (IFNB), interferon-γ (IFNG), interleukin-1β (IL1B), interleukin-2 (IL2), interleukin-6 (IL6), major histocompatibility complex class I (MHCI), major histocompatibility complex class II (MHCII), and toll-like receptor 7 (TLR7). These genes were selected for their known role in the response to AIV and their function in innate immunity.

**Figure 3:**
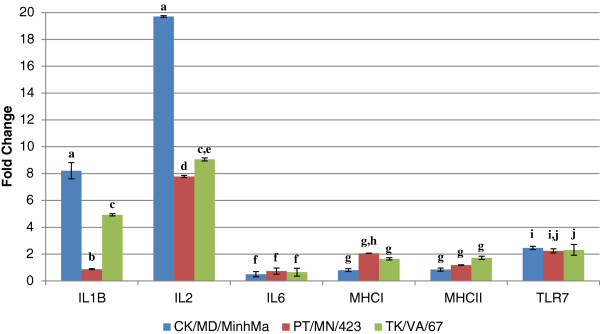
**Expression of selected cytokines and immune genes in response to infection with CK/MD/MinhMa (chicken-origin), PT/MN/423 (duck-origin), or TK/VA/67 (turkey-origin) LPAIV isolates.** qRT-PCR relative quantification results are represented as fold-change of the infected 3 d.p.i. duck spleens over the time-matched control (non-infected) duck spleen. Error bars represent standard error of the mean. Means with different letters are significantly different (Tukey-Kramer MSD, p<0.05).

Figure 3 illustrates the changes in gene expression of the interleukins, MHCs, and TLR7. IL2 demonstrated the greatest level of gene expression induction in response to all three LPAIV infections, especially during infection with CK/MD/MinhMa (19.7 fold up-regulation). IL2 was up-regulated by 7.8 and 9.1 fold for the PT/MN/423 and TK/VA/67 infections respectively. IL1B gene expression was up-regulated in response to infection with all three LPAIV isolates as well, with the greatest gene expression changes in the CK/MD/MinhMa infection at 8.2 fold. Minimal gene expression changes (<2.5 fold up-regulated) were observed for IL6, MHCI, MHCII, and TLR7.

**Figure 4:**
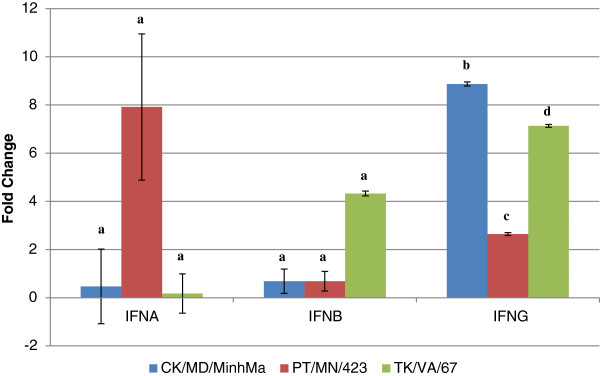
**Expression of interferon genes in response to infection with CK/MD/MinhMa (chicken-origin), PT/MN/423 (duck-origin), or TK/VA/67 (turkey-origin) LPAIV isolates.** qRT-PCR relative quantification results are represented as fold-change of the infected 3 d.p.i. duck spleens over the time-matched control (non-infected) duck spleen. Error bars represent standard error of the mean. Means with different letters are significantly different (Tukey-Kramer MSD, p<0.05).

Gene expression changes in the interferon genes are illustrated in Figure 4. The results for IFNA were not statistically significant at p<0.05, however, the results for IFNB and IFNG were statistically significant and demonstrated a 4.3 fold increase in IFNB expression in ducks infected with the turkey-origin LPAIV isolate (TK/VA/67). Large up-regulation of IFNG was seen in ducks infected with the chicken-origin isolate (8.9 fold) and in ducks infected with the turkey-origin isolate (7.1 fold).

## Discussion

In the current study, we aimed to characterize the pathogenomic host response of ducks to different species-of-origin low pathogenicity avian influenza isolates. Spackman *et al.*[3] evaluated the comparative pathogenesis of twelve isolates of H7 LPAIV on chickens, ducks, and turkeys. Specifically, they assessed pathogenesis by measuring clinical signs, viral replication titers, immunohistochemistry, and seroconversion. These methods provided insight into the pathogenesis of H7 LPAIV isolates, revealing that turkeys may be more susceptible to clinical disease than chickens or ducks, and that disease severity and the degree of virus shed was dependent on specific species and isolate combinations. To build upon the Spackman *et al.* study and investigate the molecular mechanisms of innate immunity in Pekin ducks, we utilized microarrays and qRT-PCR in order to qualify and quantify gene expression changes in response to LPAIV. Three H7 LPAI viruses were evaluated for their effect on the transcriptional activity of the duck spleen at 3 d.p.i..The three H7 LPAI viruses described in Table 1 of the Spackman *et al.* study [3] have been characterized by their isolate identification, subtype, source, hemagglutinin cleavage site, neuraminidase stalk deletion, and nonstructural gene subtype and consisted of chicken-origin A/chicken/MD/MinhMa/2004 (H7N2), duck-origin A/pintail/MN/423/1999 (H7N3), and turkey-origin A/turkey/VA/SEP-67/2002 (H7N2) LPAI viruses. For our experiment, we selected Pekin ducks that were infected with H7 LPAI viruses isolated from chickens, ducks, or turkeys, representing three different species-of-origin influenza isolates.

Based on the pathobiology of LPAI viruses, and as previously reported, ducks exhibited no clinical signs in response to LPAIV infection [3,16]. With respect to the pathology of the H7 LPAIV isolates used in this study, the highest AIV qRT-PCR titers were observed in both the OP and CL swabs of ducks in response to the duck-origin virus (PT/MN/423). OP titers were highest at 2 d.p.i. (10^4.9^ Log10 titer), while cloacal titers peaked at 4 d.p.i. (10^5.2^ Log10 titer). Both OP and CL titers remained positive through 14 d.p.i.. This finding demonstrates the adaptation of PT/MN/423 to the duck host. Given the absence of clinical signs and the limited observance of gross and microscopic lesions, the replication of AIV is indicative of active AI infection and demonstrates that ducks are managing viral pathogenesis in ways other than decreasing viral replication, suggesting they use alternate strategies to prevent disease signs.

In order to evaluate gene expression changes caused by the different species-of-origin LPAIV isolates, we utilized our avian innate immune microarray (AIIM) to characterize the transcriptomic response of ducks to LPAIV. By hybridizing RNA from infected duck spleens to our 4,959 element microarray, we were able to survey the transcriptional profiles of a critical immune organ during LPAIV infection. In general, more genes were down-regulated (1197) than up-regulated (558) (Figure 2). One hypothesis for this finding is that perhaps ducks, as asymptomatic carriers and the natural reservoir for AIV, tolerate infection due in part to down-regulation of their immune system. The overall down-regulation of immunity-related genes observed in our microarray data adds to a possible mechanistic explanation of how ducks tolerate AIV infection. In fact, disease tolerance is now being considered a distinct host defense strategy, employed by a wide variety of species [17]. Ducks may be able to fine-tune their innate immune response, differentially regulating the TLR, NOD-like receptor (NLR), cytokine-cytokine receptor interaction, and TGF-beta signaling pathways (Table 1), bypassing the negative consequences associated with AIV infection. Regardless of the origin of the LPAIV isolate, infected ducks induce both the TLR and NLR pathways, while they repress cytokine-cytokine receptor interaction pathways. Immune regulation represents one probable mechanism ducks consistently use to tolerate LPAIV infections.

AIIM data revealed two genes, JUN and PMM2, of the core set of 61 differentially expressed genes that were consistently down-regulated and found to be the most highly repressed genes (data not shown). JUN is a cellular component of the activating protein 1 (AP-1) transcription factor complex and is also a key regulator of the mitogen activated protein kinase (MAPK), influenza A, and Toll-like receptor signaling pathways [18]. JUN has also been shown to play a role in both the negative and positive regulation of viral transcription, according to the curated gene expression studies in the NextBio database (Santa Clara, CA). Recently, JUN has been demonstrated to be differentially regulated, and specifically down-regulated during LPAIV infection, in a gene expression study of avian influenza infected lung cell lines [19]. A plausible role for the down-regulation of JUN could be the host’s manipulation of its own transcriptional machinery in order to prevent tissue damage or unchecked influenza virus replication. Another gene exhibiting consistent down-regulation is phosphomannomutase 2 (PMM2), a gene found in metabolic pathways such as amino sugar and nucleotide sugar metabolism (gga00520) and fructose and mannose metabolism (gga00051) [18]. Interestingly, differential regulation of PMM2 has been associated with virus infection of chicken embryo fibroblast cell cultures, thus providing an additional line of evidence supporting PMM2 down-regulation in our study [20].

The cellular pathways activated in response to LPAIV infection in ducks confirmed an innate immune response at the transcriptional level (Table 2). Activation of the nucleotide oligomerization domain (NOD), TLR, and retinoic acid inducible gene-I (RIG-I) pathways is noteworthy, as these are primary signaling pathways in the innate immune response to AIV. Specifically, NLR signaling regulates inflammation and apoptotic cascades, while TLR signaling activates the NFKB, MAPK, and type I interferon pathways [21]. RIG-I has recently gained attention due to the fact that it is absent in chickens and present in ducks, providing a potential explanation for the differential immune responses and susceptibility between these two birds [22]. These pathways intersect at critical signaling molecules and also trigger other immune pathways (apoptosis, lymphocyte recruitment, proteolysis, MAPK signaling) and the production of interferons, cytokines, and chemokines [23], pathways and proteins critical in combating influenza infection.

An emphasis on the innate immune response of the ducks to AIV is warranted given that the strength of the innate response largely determines the strength of the subsequent adaptive immune response [24]. Additionally, it has been demonstrated that ducks lack a substantial humoral immune response to AIV [24-26], inferring an increased reliance on innate immune mechanisms. Furthermore, a robust innate immune response has been correlated to increased mean death time and decreased morbidity in Pekin ducks in response to HPAIV challenge [27].

Our qRT-PCR findings provide insight into possible host defense mechanisms in LPAIV-infected ducks. There were some overall similarities between this study and the results described by Adams *et al.*[5] with respect to disease pathogenesis and the cytokine responses of ducks to LPAIV despite the fact that our studies used different tissues (PBMC versus spleen), time points (8, 12, 36 hpi versus 72 hpi), and LPAIV subtypes (H11N9 versus H7N2 or H7N3). Specifically, the lack of clinical signs and low-level expression of IL6 is supported by the Adams *et al.*[5] study and human studies in which a positive correlation between the severity of clinical signs and IL6 plasma levels was demonstrated [28]. It was interesting to note the up-regulation of the pro-inflammatory cytokine, interleukin 2 (IL2) (Figure 3). IL2 has been implicated in the protective role of the mouse host against lethal influenza virus challenge [29] and is highly expressed in duck embryonic fibroblasts in response to HP H5N1 avian influenza infection [30]. Inferences regarding a type II interferon response can also be made since the type II interferon (IFNG) was up-regulated in response to the chicken- and turkey-origin LPAIV isolates (8.9 and 7.1 fold, respectively) (Figure 4). IFNG up-regulation has been demonstrated in duck PBMC in response to a duck-origin H11N9 LPAIV infection [5]. Taken together, these results point to a type II-mediated IFN response that ducks utilize to combat LPAIV infections caused by isolates that are not duck-origin. Modulation of these critical innate immune genes provides further evidence of duck immune system fine-tuning of the innate immune response to different isolates of H7 LPAIV.

## Conclusions

In conclusion, we have identified several immune pathways that are activated in response to LPAIV infection of ducks. While many of these pathways have been previously associated with influenza virus infection, this study identified new cellular pathways associated LPAIV infected ducks, such as the fructose and mannose metabolism (gga00051) and amino sugar and nucleotide sugar metabolism (gga00520) pathways. Additionally, we have gained further insight into the differences and similarities among innate immune responses based on the avian species from which the LPAIV was isolated. A core set of 61 genes was differentially expressed during all three LPAIV infections while 101, 135, and 628 genes were uniquely differentially expressed in response to the chicken-, duck-, and turkey-origin isolates respectively, indicating the importance of host-adaptation of LPAIV on transcriptional immune responses. Further studies will be required to elucidate the virus and host mechanisms controlling gene expression during infection and to understand what factors contribute to the differential host immune response.

## Methods

### Viruses

Three H7 LPAI viruses were selected to represent different species of origin (Table 3). Viruses were propagated and titrated in 9 to 11 day-old embryonated chicken eggs by standard procedures [31]. The chicken-origin isolate (A/chicken/Maryland/MinhMa/2004) was described in 2004 by Ladman *et al.*[32] during an outbreak in 6-wk-old commercial broilers on the Minh Ma Farm in Wicomico County, Maryland. The duck-origin isolate (A/pintail/Minnesota/423/1999) was described in 2005 by Spackman *et al.*[33] during an evaluation of North American AIV natural reservoirs (free-flying waterfowl). The turkey-origin isolate (A/turkey/Virginia/SEP-67/2002) was described in 2002 by Spackman *et al.*[34] during a commercial turkey farm outbreak in Virginia, West Virginia, and North Carolina.

**Table 3:** Low pathogenicity avian influenza virus isolates evaluated for pathogenesis in Pekin ducks

**Isolate**	**Subtype**	**Source**	**Abbreviation**
A/chicken/Maryland/MinhMa/2004	H7N2	Broiler chickens*	CK/MD/MinhMa
A/pintail/Minnesota/423/1999	H7N3	Wild Pintail ducks	PT/MN/423
A/turkey/Virginia/SEP-67/2002	H7N2	Meat-type turkeys*	TK/VA/67

*Live bird market (LBM) lineage.

### Animals

Pekin ducks (*Anas platyrhynchos domesticus*) were obtained from commercial hatcheries at day of age and were housed in negative pressure glove-port isolators (Allentown Caging, Allentown, NJ) under biosafety level 3 containment conditions in the Charles C. Allen Biotechnology Laboratory at the University of Delaware. Ducks were obtained from flocks with no antibody or prior exposure to AI virus. The ducks were provided with *ad libitum* access to feed and water before and after exposure to the viruses. Ducks were cared for in accordance with established humane procedures and University of Delaware biosecurity guidelines.

### Evaluation of viral pathogenicity in pekin ducks

Fifteen Pekin ducks were separated into four treatment groups: Group 1 – Non-infected controls, Group 2 - CK/MD/MinhMa inoculated, Group 3 - PT/MN/423 inoculated, and Group 4 - TK/VA/67 inoculated. At 2 weeks of age, each duck was inoculated with 10^6^ EID_50_ per bird in 0.1 ml by the intrachoanal (cleft palate) route. Birds were monitored daily for clinical disease signs which were scored as follows: 0 = no clinical signs, 1 = mild depression, 2 = moderate to severe (i.e. depressed, not eating, neurological signs), 3 = dead. Oral-pharyngeal (OP) and cloacal (CL) swabs were collected at 2, 4, 7, 10 and 14 days post-inoculation (d.p.i.) to evaluate virus shed by quantitative real-time RT-PCR (qRT-PCR) [3]. Three d.p.i., 3 birds from each treatment group were euthanized and necropsied to evaluate gross lesions and collect spleens. One hundred mg of spleen tissue was collected from each bird, and stored in 5–10 volumes of RNAlater at −80°C for RNA isolation and subsequent microarray and qRT-PCR analysis.

### RNA isolation

Spleen samples from each of the three birds selected for necropsy were pooled according to treatment group. Total cellular RNA was isolated from 100 mg of spleen tissue using the RNeasy Midi RNA Purification Kit (Qiagen Inc., Valencia, CA) according to the manufacturer’s protocols. The optional DNaseI on-column digestion step was employed to remove any trace or contaminating duck genomic DNA from the samples. RNA quantity was determined using a Nanodrop 1000 (Nanodrop, Wilmington, DE), and RNA quality was assessed using the Agilent RNA 6000 Nano Assay Protocol in the Agilent 2100 Bioanalyzer (Agilent Technologies, Santa Clara, CA). RNA Integrity Numbers (RINs) were obtained for each sample to confirm sample quality.

### RNA amplification, fluorescent labeling, and hybridization

One μg of total cellular RNA from each treatment group pool was amplified into amino allyl modified RNA (aRNA) using the Ambion Amino Allyl MessageAmp II aRNA Amplification Kit (Ambion Inc., Austin, TX) using two rounds of amplification and following the manufacturer’s instructions. Ten ug of aRNA mixed with 9 μL of coupling buffer was fluorescently labeled with Alexa Fluor 555 (Invitrogen, Carlsbad, CA) and resuspended in 11 μL of DMSO. The labeling reaction was performed at room temperature for 3 hours in the dark. Post-labeling aRNA purification, post-hybridization washes, and microarray slide scanning were performed as previously described [4] and hybridization to the AIIM was conducted at 42°C overnight.

### Microarray data analysis

Spot and background intensities were acquired using GenePix Pro 4.1 Software (Molecular Devices, Sunnyvale, CA). Abnormal spots (dust, bubbles in the hybridization solution) were removed from further analysis. Spot intensity was determined using a local background subtraction method. Data from analyzed slides was imported to GeneSpring v7.3 (Agilent Technologies, Santa Clara, CA). Each experimental slide was compared to the control slide (non-infected duck spleen) to determine relative spot intensities, and differential gene expression. A gene list was created from those elements that appeared in two of the three replicate spot locations in each slide, in all three experimental conditions (i.e. infections with either the chicken-, duck-, or turkey- species-of-origin LPAI isolates). Subsets of this gene list consisting of two-fold differentially regulated genes from each infected treatment group were exported for further pathway and gene ontology (GO) analysis. Lists of differentially expressed genes were created using GeneSpring v7.3. The corresponding Entrez Gene IDs were imported to AgBase v2.0 GORetriever to obtain GO IDs [35]. The GORetriever GO ID output was then analyzed in The Database for Annotation, Visualization and Integrated Discovery (DAVID) v6.7 [36,37]. Functional annotation and gene functional annotation analyses were performed using DAVID, which provided batch annotation and GO term enrichment analysis to highlight the most relevant GO terms associated with the input gene list. Further DAVID analysis yielded the significant Kyoto Encyclopedia of Genes and Genomes (KEGG) pathways represented in the data set.

### Quantitative real-time RT-PCR (qRT-PCR)

qRT-PCR targeting select duck immune genes was performed on the splenic RNA samples (Table 4). Primer sequences were kindly provided by Dr. Darrell Kapczynski (DK, personal communication) and Dr. Carol Cardona as referenced. qRT-PCR was performed with aliquots of RNA from the same samples that were used in the AIIM microarray analysis. Gene expression levels of mRNA transcripts were determined by qRT-PCR using a QuantiTect SYBR Green RT-PCR kit (Qiagen). qRT-PCR was performed for each sample in triplicate on an ABI 7900HT Sequence Detection System (Life Technologies Corp., Carlsbad, CA). The amplification procedure was performed in a 20 μL reaction volume containing 300 nM of each primer and 100 ng of RNA. The following thermal-cycling conditions were used: reverse transcription (30 min at 50°C), PCR initial activation (15 min at 95°C), and 40 cycles of denaturation (15 sec at 94°C), annealing (30 sec at 55°C), and extension (30 sec at 72°C). Data were analyzed using SDS2.3 (Life Technologies Corp.).

**Table 4:** Real-time quantitative RT-PCR primers

**RNA target**	**Forward primer (5’ – 3’)**	**Reverse primer (5’ – 3’)**	**Genbank accession #**	**Ref.**
IFNA	GACAGCCAACGCCAAAGC	AATGCTTGAGCAGCAGCGAC	EF053034	(5)
IFNB	CCTCAACCAGATCCAGCATT	GGATGAGGCTGTGAGAGGAG	AY831397	(5)
IFNG	CAACGCTCAACTACTCTC	TGTGGTTAATCTGTCCTTAG	AJ012254	DK
IL1B	TCGACATCAACCAGAAGTGC	GAGCTTGTAGCCCTTGATGC	DQ393268	(5)
IL2	GCCAAGAGCTGACCAACTTC	ATCGCCCACACTAAGAGCAT	AF294323	(5)
IL6	TTCGACGAGGAGAAATGCTT	CCTTATCGTCGTTGCCAGAT	AB191038	(5)
MHCI	GAAGGAAGAGACTTCATTGCCTTGG	CTCTCCTCTCCAGTACGTCCTTCC	AB115246	(5)
MHCII	CCACCTTTACCAGCTTCGAG	CCGTTCTTCATCCAGGTGAT	AY905539	(5)
TLR7	CCTTTCCCAGAGAGCATTCA	TCAAGAAATATCAAGATAATCACATCA	AY940195	(5)
GAPDH	ATGTTCGTGATGGGTGTGAA	CTGTCTTCGTGTGTGGCTGT	AY436595	(5)

### qRT-PCR data and statistical analysis

Average cycle threshold (Ct) values for each target gene were normalized by the Ct value of an endogenous control gene, glyceraldehyde-3-phosphate dehydrogenase (GAPDH). Relative gene expression data were analyzed using the Livak and Schmittgen 2^-ΔΔCt^ method [38] and ΔCt values were calculated by subtracting average GAPDH Ct values from average target gene Ct values. Normalized Ct values (ΔCt) from LPAIV infected samples was compared to the ΔCt from non-infected control duck spleen samples, the difference (ΔΔCt) being transformed into 2^-ΔΔCt^ value as the estimated fold change of the experimental sample (infected) over the control (non-infected) sample. The three replicate Ct values for each gene were analyzed by one-way ANOVA (p<0.05) to determine the statistical significance between means of individual genes. A post-hoc statistical test, Tukey-Kramer minimum significant differences (MSD), was utilized to analyze the differences amongst means of genes grouped by LPAIV isolate (p<0.05).

## Abbreviations

LPAI: Low pathogenicity avian influenza; H: Hemagglutinin; AI: Avian influenza; AIV: Avian influenza virus; LP: Low pathogenicity; HP: High pathogenicity; AIIM: Avian innate immunity microarray; LPAIV: Low pathogenicity avian influenza; PBMC: Peripheral blood mononuclear cells; d.p.i.: Days post-infection; qRT-PCR: quantitative real-time RT-PCR; OP: Oral-pharyngeal; CL: Cloacal; GO: Gene ontology; KEGG: Kyoto Encyclopedia of Genes and Genomes; DAVID: Database for Annotation, Visualization and Integrated Discovery; JUN: Jun oncogene; PMM2: Phosphomannomutase 2; IFNA: Interferon-α; IFNB: Interferon-β; IFNG: Interferon-γ; IL1B: Interleukin-1β; IL2: Interleukin-2; IL6: Interleukin-6; MHCI: Major histocompatibility complex class I; MHCII: Major histocompatibility complex class II; TLR7: Toll-like receptor 7; NLR: NOD-like receptor; AP-1: Activating protein 1; MAPK: Mitogen activated protein kinase; NOD: Nucleotide oligomerization domain; RIG-I: Retinoic acid inducible gene-I; aRNA: Amino allyl modified RNA; Ct: Cycle threshold; GAPDH: Glyceraldehyde-3-phosphate dehydrogenase; MSD: Minimum significant differences; DK: Darrell Kapczynski.

## Competing interests

The authors declare that they have no competing interests.

## Author contributions

ES was involved in virus selection and experimental design. JG was involved in virus selection and experimental design. BL and LP conducted animal experiments, collected specimens, ran qRT-PCR, and serological assays. CK, LD, and MM designed the microarray and qRT-PCR experiments targeting immune genes. LD and MM performed the microarray experiments. MM performed the qRT-PCR data and statistical analysis. All authors contributed to manuscript preparation. All authors read and approved the final manuscript.
